# Treatment of axial spondyloarthritis with biologic and targeted synthetic DMARDs: British Society for Rheumatology guideline scope

**DOI:** 10.1093/rap/rkad039

**Published:** 2023-05-15

**Authors:** Sizheng Steven Zhao, Stephanie R Harrison, Antoni Chan, Nick Clarke, Charlotte Davis, Joe Eddison, William J Gregory, Gareth T Jones, Helena Marzo-Ortega, Daniel J Murphy, Virinderjit Sandhu, Raj Sengupta, Stefan Siebert, Ben Thompson, Dale Webb, Max Yates, Karl Gaffney

**Affiliations:** Centre for Epidemiology Versus Arthritis, Division of Musculoskeletal and Dermatological Science, School of Biological Sciences, Faculty of Biological Medicine and Health, The University of Manchester, Manchester Academic Health Science Centre, Manchester, UK; Leeds Institute of Rheumatic and Musculoskeletal Medicine, University of Leeds, Leeds, UK; NIHR Leeds Biomedical Research Centre, Leeds Teaching Hospitals Trust, Leeds, UK; Department of Rheumatology, Royal Berkshire NHS Foundation Trust, Reading, UK; Patient Expert; Department of Rheumatology, Leeds Teaching Hospitals Trust, UK; Patient Expert; Rheumatology Department, Salford Royal Hospital, Northern Care Alliance NHS Foundation Trust, Greater Manchester, UK; Faculty of Health and Education, Manchester Metropolitan University, Manchester, UK; Aberdeen Centre for Arthritis and Musculoskeletal Health (Epidemiology Group), University of Aberdeen, Aberdeen, UK; Leeds Institute of Rheumatic and Musculoskeletal Medicine, University of Leeds, Leeds, UK; NIHR Leeds Biomedical Research Centre, Leeds Teaching Hospitals Trust, Leeds, UK; Department of Rheumatology, Honiton Surgery, Royal Devon & Exeter Hospital, Exeter, UK; Department of Rheumatology, St George’s University Hospitals NHS Foundation Trust, London, UK; Royal National Hospital for Rheumatic Diseases, Royal United Hospitals, Bath, UK; School of Infection and Immunity, University of Glasgow, Glasgow, UK; Rheumatology Department, The Newcastle-upon-Tyne Hospitals NHS Foundation Trust, Newcastle-upon-Tyne, UK; National Axial Spondyloarthritis Society (NASS), London, UK; Centre for Epidemiology, Norwich Medical School, University of East Anglia, Norwich, UK; Rheumatology Department, Norfolk & Norwich University Hospitals NHS Foundation Trust, Norwich, UK; Rheumatology Department, Norfolk & Norwich University Hospitals NHS Foundation Trust, Norwich, UK

**Keywords:** Axial spondyloarthritis, AS, biologic, biosimilar, IL17, JAK inhibitor, treat-to-target, switching, tapering

## Abstract

Pharmacological management has advanced considerably since the 2015 British Society for Rheumatology axial spondyloarthritis (axSpA) guideline to incorporate new classes of biologic DMARDs (bDMARDs, including biosimilars), targeted synthetic DMARDs (tsDMARDs) and treatment strategies such as drug tapering. The aim of this guideline is to provide an evidence-based update on pharmacological management of adults with axSpA (including AS and non-radiographic axSpA) using b/tsDMARDs. This guideline is aimed at health-care professionals in the UK who care directly for people with axSpA, including rheumatologists, rheumatology specialist nurses, allied health professionals, rheumatology specialty trainees and pharmacists; people living with axSpA; and other stakeholders, such as patient organizations and charities.


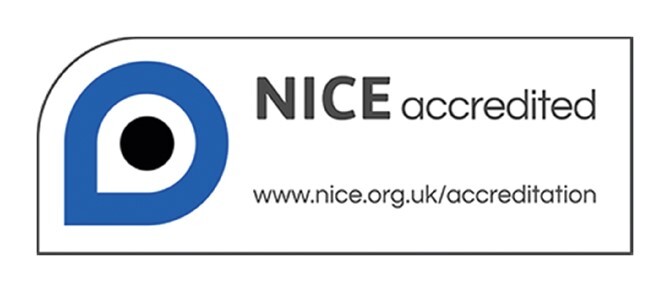


NICE has accredited the process used by BSR to create its clinical guidelines. The term began on 27 February 2012 and the current renewed accreditation is valid until 31 December 2023. More information on accreditation can be viewed at www.nice.org.uk/accreditation.

## Why the guideline is needed

Since the 2015 BSR and British Health Professionals in Rheumatology treatment guideline for axial spondyloarthritis (axSpA) [2], pharmacological management has advanced considerably to incorporate new classes of biologic DMARDs (bDMARD, including biosimilars), targeted synthetic DMARDs (tsDMARD) and treatment strategies such as drug tapering. An updated BSR guideline is needed to inform health-care providers and other stakeholders. Although European and North American societies have both recently published treatment guidelines (Assessment of SpondyloArthritis international Society/European Alliance of Associations for Rheumatology [3] and ACR/Spondyloarthritis Research and Treatment Network/Spondylitis Association of America [4]), they are not always directly transferable or applicable to the health-care system in the UK. For example, drugs may receive authorization at different times across health-care systems. The publicly funded health-care system in the UK may allocate resources differently, with implications for availability and use of licensed drugs. Because of the higher costs associated with these treatments, prescribing in England, Wales and Northern Ireland comes under the guidance of the National Institute for Health and Care Excellence (NICE), and in Scotland the Scottish Medicines Consortium.

## Key facts and figures

AxSpA is a chronic inflammatory disease that predominantly affects the spine and sacroiliac joints [5]. It can also involve peripheral joints and entheses, and extra-musculoskeletal manifestations such as acute anterior uveitis, psoriasis and IBD. The axSpA disease spectrum can be classified into those who have developed structural damage in the sacroiliac joints visible on radiographs (AS or radiographic axSpA) and those without such damage (non-radiographic axSpA). Clinical features, symptom severity, co-morbidities and treatment response are comparable between radiographic and non-radiographic groups [6, 7].

Symptoms of axSpA typically start in early adulthood, but diagnosis can take several years. Chronic inflammatory pain and stiffness are well recognized to have adverse effects on quality of life, social participation and mental health [8–10]. The co-morbidity burden is also higher than in age-matched people without axSpA [11], which can influence treatment choice.

## Current practice

The key aims of axSpA management are to control symptoms, restore function and quality of life, and slow disease progression [2, 3, 12]. Optimal management should be holistic, addressing musculoskeletal and extra-musculoskeletal manifestations as well as co-morbidities, and should include both pharmacological and non-pharmacological approaches. Multidisciplinary care is essential.

Non-pharmacological modalities (e.g. physiotherapy, hydrotherapy, lifestyle interventions and patient education) form the cornerstone of management. Randomized controlled trials of non-pharmacological interventions can be methodologically challenging, and limited evidence has emerged beyond those reviewed previously [13, 14]. Therefore, the current guideline update will focus on pharmacological management only, specifically on developments in b/tsDMARDs (collectively referred to as targeted therapies henceforth). To ensure that our UK guideline appropriately profiles the breadth of treatment required for axSpA, a summary will be provided of the non-pharmacological management recommendations from recently published guidelines from European and North American societies [3, 4].

Pharmacological management generally starts with NSAIDs and, if symptom control remains inadequate, escalation to targeted therapies may be indicated. Up to half of patients starting their first bDMARD do not respond adequately [15, 16], the reasons for which are not completely understood. Unlike other inflammatory arthritides, such as RA and PsA, the number of pharmacological treatment options in axSpA is comparably limited, comprising inhibitors of TNF, IL17 and the Janus kinases (JAK).

## Who the guideline is for

This guideline is for health professionals in the UK who care directly for people with axSpA, including rheumatologists, rheumatology specialist nurses, allied health professionals, rheumatology specialty trainees, pharmacists; people living with axSpA; and other stakeholders, such as patient organizations and charities.

## What the guideline will cover

### Target clinical population

Adults with axSpA, including AS (i.e. radiographic axSpA) and non-radiographic axSpA.

### Settings

Secondary/tertiary care rheumatology (targeted therapies are restricted to specialist use).

### Activities, services or aspects of care

Key areas that will be covered:

- Pharmacological treatment of people with axial spondyloarthritis using b/tsDMARDs, including biosimilars.
- Treatment strategies including switching, tapering, withdrawal and treat-to-target approaches.

Areas that will not be covered:

- Treatment of enthesitis- or spondylitis-related JIA.
- Axial disease in PsA.
- NSAIDs, glucocorticoids and conventional synthetic DMARDs.
- Non-pharmacological management (a brief summary from related guidelines will be included).

Related guidance:

- BSR and BHPR guideline for the treatment of axSpA (including AS) with biologics [2].
- ASAS–EULAR recommendations for the management of axSpA: 2022 update [3].
- 2019 Update of the American College of Rheumatology/Spondylitis Association of America/Spondyloarthritis Research and Treatment Network recommendations for the treatment of AS and non-radiographic axSpA [4].
- NICE guideline [NG65] Spondyloarthritis in over 16s: diagnosis and management [17].
- Development of ASAS quality standards to improve the quality of health and care services for patients with axSpA [12].
- BSR guideline on prescribing drugs in pregnancy and breastfeeding: immunomodulatory anti-rheumatic drugs and corticosteroids [18].
- 2022 EULAR recommendations for screening and prophylaxis of chronic and opportunistic infections in adults with autoimmune inflammatory rheumatic diseases [19].
- The 2022 BSR guideline for the treatment of PsA with biologic and tsDMARDs [20].

## Key issues and draft questions

We identified the following draft questions, which will be used to develop more detailed review questions and methodology. Where indicated, we will evaluate evidence from both clinical trials and real-world observational studies. It might not be possible to make recommendations in all areas. Targeted therapies refer to bDMARDs (including biosimilars) and tsDMARDs, including inhibitors of TNF, IL17 and JAK.

- In adults with active axSpA, what is the clinical effectiveness and safety of targeted therapies, compared to each other or placebo, on:
  - Axial symptoms and manifestations;
  - Peripheral musculoskeletal manifestations, namely, arthritis, dactylitis and enthesitis;
  - Extra-musculoskeletal manifestations, namely, acute anterior uveitis, psoriasis and IBD;
  - Co-morbidities and risk factors (including the impact of co-morbidities or risk factors on choice of targeted therapy and effect of therapy on common co-morbidities)?
- In adults with active axSpA who do not respond adequately to or tolerate one or more targeted therapies, what is the clinical effectiveness and safety of switching:
  - to biosimilars;
  - to targeted therapies with different mechanisms of action;
  - after multiple targeted therapies?
- In adults with active axSpA, what is the clinical effectiveness and safety of combining targeted therapies (including those licensed for extra-musculoskeletal manifestations)?
- In adults with active axSpA, what is the evidence for a treat-to-target strategy compared with usual care?
- In adults with axSpA who have achieved clinical remission or low disease activity, what is the evidence, compared with usual care, for:
  - tapering or dose reduction of targeted therapies;
  - withdrawing targeted therapies;
  - switching to biosimilars?

## Guideline working group constituency

Karl Gaffney (co-chair), rheumatologist

Sizheng Steven Zhao (co-chair), clinical lecturer in rheumatology

Antoni Chan, rheumatologist

Stephanie Harrison, rheumatology fellow

Helena Marzo-Ortega, rheumatologist

Virinderjit Sandhu, rheumatologist

Raj Sengupta, rheumatologist

Stefan Siebert, rheumatologist

Ben Thompson, rheumatologist

Max Yates, rheumatologist

Nick Clarke, patient expert

Joe Eddison, patient expert

Dale Webb, National Axial Spondyloarthritis Society

William Gregory, consultant physiotherapist

Gareth Jones, epidemiologist

Daniel Murphy, general practitioner

Charlotte Davis, rheumatology specialist nurse

## Data Availability

No new data were generated in support of this work.

## References

[1] British Society for Rheumatology. Creating Clinical Guidelines Protocol v.5.3. Revised on behalf of SAGWG. 2022. Internal company document (unpublished).

[2] Hamilton L , Barkham N, Bhalla A et al; BSR and BHPR Standards, Guidelines and Audit Working Group. BSR and BHPR guideline for the treatment of axial spondyloarthritis (including ankylosing spondylitis) with biologics. Rheumatology (Oxford) 2017;56:313–6. 27558584 10.1093/rheumatology/kew223

[3] Ramiro S , Nikiphorou E, Sepriano A et al ASAS-EULAR recommendations for the management of axial spondyloarthritis: 2022 update. Ann Rheum Dis 2023;82:19–34. 36270658 10.1136/ard-2022-223296

[4] Ward MM , Deodhar A, Gensler LS et al 2019 update of the American Sollege of Rheumatology/Spondylitis Association of America/Spondyloarthritis Research and Treatment Network recommendations for the treatment of ankylosing spondylitis and nonradiographic axial spondyloarthritis. Arthritis Rheumatol 2019;71:1599–613. 31436036 10.1002/art.41042 PMC6764882

[5] Sieper J , Poddubnyy D. Axial spondyloarthritis. Lancet 2017;390:73–84. 28110981 10.1016/S0140-6736(16)31591-4

[6] Michelena X , Zhao SS, Dubash S et al Similar biologic drug response regardless of radiographic status in axial spondyloarthritis: data from the British Society for Rheumatology Biologics Register in Ankylosing Spondylitis registry. Rheumatology (Oxford) 2021;60:5795–800. 33502476 10.1093/rheumatology/keab070 PMC8645273

[7] Zhao SS , Ermann J, Xu C et al Comparison of comorbidities and treatment between ankylosing spondylitis and non-radiographic axial spondyloarthritis in the United States. Rheumatology (Oxford) 2019;58:2025–30. 31081033 10.1093/rheumatology/kez171 PMC7967894

[8] Hollick RJ , Stelfox K, Dean LE et al Outcomes and treatment responses, including work productivity, among people with axial spondyloarthritis living in urban and rural areas: a mixed-methods study within a national register. Ann Rheum Dis 2020;79:1055–62. 32522742 10.1136/annrheumdis-2020-216988 PMC7392479

[9] Macfarlane GJ , Rotariu O, Jones GT, Pathan E, Dean LE. Determining factors related to poor quality of life in patients with axial spondyloarthritis: results from the British Society for Rheumatology Biologics Register (BSRBR-AS). Ann Rheum Dis 2020;79:202–8. 31662321 10.1136/annrheumdis-2019-216143

[10] Zhao S , Thong D, Miller N et al The prevalence of depression in axial spondyloarthritis and its association with disease activity: a systematic review and meta-analysis. Arthritis Res Ther 2018;20:140. 29996916 10.1186/s13075-018-1644-6 PMC6042424

[11] Zhao SS , Robertson S, Reich T et al Prevalence and impact of comorbidities in axial spondyloarthritis: systematic review and meta-analysis. Rheumatology (Oxford) 2020;59:iv47–iv57. 33053193 10.1093/rheumatology/keaa246 PMC7566561

[12] Kiltz U , Landewé RBM, van der Heijde D et al Development of ASAS quality standards to improve the quality of health and care services for patients with axial spondyloarthritis. Ann Rheum Dis 2020;79:193–201. 31604704 10.1136/annrheumdis-2019-216034 PMC7025729

[13] Dagfinrud H , Kvien TK, Hagen KB. Physiotherapy interventions for ankylosing spondylitis. Cochrane Database Syst Rev 2008;2008:CD002822. 18254008 10.1002/14651858.CD002822.pub3 PMC8453259

[14] Ortolan A , Webers C, Sepriano A et al Efficacy and safety of non-pharmacological and non-biological interventions: a systematic literature review informing the 2022 update of the ASAS/EULAR recommendations for the management of axial spondyloarthritis. Ann Rheum Dis 2023;82:142–52. 36261247 10.1136/ard-2022-223297

[15] Zhao SS , Jones GT, Macfarlane GJ et al Comorbidity and response to TNF inhibitors in axial spondyloarthritis: longitudinal analysis of the BSRBR-AS. Rheumatology (Oxford) 2021;60:4158–65. 33369676 10.1093/rheumatology/keaa900 PMC8409999

[16] Lord PA , Farragher TM, Lunt M et al; BSR Biologics Register. Predictors of response to anti-TNF therapy in ankylosing spondylitis: results from the British Society for Rheumatology Biologics Register. Rheumatology (Oxford) 2010;49:563–70. 20032223 10.1093/rheumatology/kep422 PMC2820265

[17] National Institute for Health and Care Excellence (NICE). Guideline [NG65] Spondyloarthritis in over 16s: diagnosis and management. 2017. https://www.nice.org.uk/guidance/ng65 (26 January 2023, date last accessed). 32049469

[18] Russell MD , Dey M, Flint J et al British Society for Rheumatology guideline on prescribing drugs in pregnancy and breastfeeding: immunomodulatory anti-rheumatic drugs and corticosteroids. Rheumatology (Oxford) 2022;keac551. 10.1093/rheumatology/keac551 PMC10070073 36318966

[19] Fragoulis GE , Nikiphorou E, Dey M et al 2022 EULAR recommendations for screening and prophylaxis of chronic and opportunistic infections in adults with autoimmune inflammatory rheumatic diseases. Ann Rheum Dis 2022;ard-2022-223335. 10.1136/ard-2022-223335 36328476

[20] Tucker L , Allen A, Chandler D et al The 2022 British Society for Rheumatology guideline for the treatment of psoriatic arthritis with biologic and targeted synthetic DMARDs. Rheumatology (Oxford) 2022;61:e255–66. 35640657 10.1093/rheumatology/keac295

